# The novel CXCR4 antagonist POL5551 mobilizes hematopoietic stem and progenitor cells with greater efficiency than Plerixafor

**DOI:** 10.1038/leu.2013.266

**Published:** 2013-09-27

**Authors:** D Karpova, K Dauber, G Spohn, D Chudziak, E Wiercinska, M Schulz, A R Pettit, J P Levesque, B Romagnoli, K Patel, E Chevalier, K Dembowsky, H Bonig

**Affiliations:** 1 German Red Cross Blood Service, Institute for Transfusion Medicine and Immunohematology of the Goethe University, Frankfurt, Germany; 2 UQ Centre for Clinical Research, The University of Queensland, Herston, Queensland, Australia; 3 Mater Research Institute - University of Queensland, Translational Research Institute, Woolloongabba, Queensland, Australia; 4 Polyphor Ltd, Allschwil, Switzerland; 5 Department of Medicine/Hematology, University of Washington, Seattle, WA, USA

**Keywords:** mobilization, CXCR4, CXCL12, G-CSF, hematopoietic stem and progenitor cells

## Abstract

Mobilized blood has supplanted bone marrow (BM) as the primary source of hematopoietic stem cells for autologous and allogeneic stem cell transplantation. Pharmacologically enforced egress of hematopoietic stem cells from BM, or mobilization, has been achieved by directly or indirectly targeting the CXCL12/CXCR4 axis. Shortcomings of the standard mobilizing agent, granulocyte colony-stimulating factor (G-CSF), administered alone or in combination with the only approved CXCR4 antagonist, Plerixafor, continue to fuel the quest for new mobilizing agents. Using Protein Epitope Mimetics technology, a novel peptidic CXCR4 antagonist, POL5551, was developed. *In vitro* data presented herein indicate high affinity to and specificity for CXCR4. POL5551 exhibited rapid mobilization kinetics and unprecedented efficiency in C57BL/6 mice, exceeding that of Plerixafor and at higher doses also of G-CSF. POL5551-mobilized stem cells demonstrated adequate transplantation properties. In contrast to G-CSF, POL5551 did not induce major morphological changes in the BM of mice. Moreover, we provide evidence of direct POL5551 binding to hematopoietic stem and progenitor cells (HSPCs) *in vivo*, strengthening the hypothesis that CXCR4 antagonists mediate mobilization by direct targeting of HSPCs. In summary, POL5551 is a potent mobilizing agent for HSPCs in mice with promising therapeutic potential if these data can be corroborated in humans.

## Introduction

Mobilization of hematopoietic stem and progenitor cells (HSPCs) describes their enforced egress from the bone marrow (BM), their natural place of residence in post-natal mammals, into the peripheral blood (PB). HSPC mobilization occurs in response to a wide variety of physiological or pharmacological stimuli, such as intense physical exercise, infection or inflammation, and administration of cytokines or chemotherapy.^1, 2, 3^ The clinically most relevant mobilizing agent, granulocyte colony-stimulating factor^4, 5^ (G-CSF), promotes mobilization by a complex chain of indirect convergent cellular and molecular events including interference with the CXCL12/CXCR4 axis.^6, 7^ The 5-day course of G-CSF stimulation required for optimal HSPC mobilization^5, 8, 9^ results in substantial variability in mobilization efficiency.^10^ Added to the adverse effects of G-CSF,^11, 12, 13, 14, 15^ such as significant BM disruption^16, 17, 18^ and the lingering threat of adverse genetic events induced by G-CSF,^19, 20^ these shortcomings have driven the quest for alternative mobilizing agents devoid of some of these inherent disadvantages. Direct targeting of CXCR4 with small molecule antagonists has been used to mobilize HSPCs, most prominently with the bicyclam antagonist Plerixafor.^21, 22, 23, 24^ However, CXCR4 inhibitors available to date have proven too weak for efficient clinical mobilization when given as a single agent.^22, 24^

CXCR4-deficient hematopoiesis is characterized by a severe HSPC retention defect in the BM that manifests as constitutive mobilization.^25^ This phenotype suggests that the cellular target of CXCR4 antagonists that results in HSPC egress from marrow is the HSPC proper. Indeed, this mechanism has been assumed by many;^22, 26, 27^ however, direct evidence of this hypothesis has been lacking and recently published data potentially challenge this notion.^28^

We here report on a novel, potent and highly selective CXCR4 antagonist, POL5551, which was developed using the Protein Epitope Mimetics technology.^29^ Using *in vitro* and *in vivo* assays, we explored in mice the potential of POL5551 as an HSPC-mobilizing agent. Using labeled compound, we also sought to identify the cellular target of CXCR4 antagonist-mediated mobilization.

## Materials and methods

### Mice

C57BL/6 wild-type (CD45.2) mice purchased from Janvier (Le Genest-Saint-Isle, France) or Charles River Laboratories (Sulzfeld, Germany) were used for most experiments. B6.SJL-*Ptprc*^*a*^*Pep3*^*b*^/BoyJ (CD45.1, Charles River Laboratories) and F1-hybrid mice (CD45.1/2) were used for engraftment experiments. B6.SJL-*Ptprc*^*a*^*Pep3*^*b*^/BoyJ and DBA/2 mice (Janvier) were used for some mobilization experiments. Animals were housed at the Johann Wolfgang Goethe-University Medical School vivarium under non-SPF conditions, with autoclaved chow and water *ad libitum*. Following lethal irradiation (1 × 9.5 Gy, except for homing assays, where 1 × 12.5 Gy were used, using a Cesium source with a dose rate of 1 Gy/min) and transplantation, mice were kept on antibiotic medication, 0.025% Baytril (Bayer, Leverkusen, Germany) p.o. in drinking water. All procedures were approved by the municipal government (Darmstadt, Germany) and the institutional animal care and use committee, in agreement with the Association for Assessment and Accreditation of Laboratory Animal Care (AAALAC) guidelines.

### Cells

The murine Ao.o1 T cell line,^30^ a kind gift from Dr Françoise Bachelerie (Unité d'Immunologie Virale, Institut Pasteur, Paris, France), was engineered to overexpress human CXCR4 under a retroviral promoter. For details see Supplementary Methods. The generated cell line will be referred to as Ao.o1_hCXCR4 throughout the manuscript.

PB was drawn from the facial vein of the mice. BM cells were recovered by flushing femurs, tibias or pelvic bones. Spleen cells were isolated by gentle blunt extrusion from the capsule. For most of the *in vitro* studies (migration, F-actin polymerization, flow cytometry, colony assay) as well as for the homing assay, cells were washed and erythrocytes were lysed with ammonium chloride lysis buffer (Sigma-Aldrich, St Louis, MO, USA; or BD Biosciences, San Jose, CA, USA) prior to the assay performance.

### Fluorescence-activated cell sorting and analysis

Cell labeling was performed according to standard protocols using established marker panels for identification of different subsets in mouse hematopoietic tissues. Antibodies used in this study are detailed in Supplementary Methods. Subsequent acquisition and analysis were performed on a BD FACSCanto II cytometer with the FACSDiva software (BD Biosciences). Some data were further analyzed using the FlowJo software (Tree Star, Inc., Ashland, OR, USA). Cell isolation by flow sorting was performed on a BD FACS Aria II (BD Biosciences).

### Receptor binding studies

Ao.o1_hCXCR4 cells (see above) were used to study occupation of different receptor domains by the natural ligand of CXCR4, CXCL12, in comparison to the antagonists Plerixafor and POL5551. A total of 1–2 × 10^5^ cells were concurrently incubated with CXCL12, Plerixafor or POL5551 (1 μM in phosphate-buffered saline (PBS)/bovine serum albumin, 0.5%, for all) and one of the two different CXCR4 antibody clones 12G5 (binding to extracellular loops) or 1D9 (binding to the N-terminus). Controls were incubated with the antibodies alone or stained with appropriate immunoglobulin G isotype controls. Incubation was performed at 4 °C (to prevent internalization) in the dark for 30 min followed by a wash step and fluorescence-activated cell sorting analysis of the samples.

### Migration

Migration of BM or PB cells through 5-μm pore-size transwells (Corning-Costar, Tewksbury, MA, USA) towards CXCL12 (100 ng/ml, Peprotech, Rocky Hill, NJ, USA or Cell Systems, Kirkland, WA, USA), or control medium (spontaneous migration), performed as described,^23^ was assessed after 4 h. Input cells and cells from the lower chamber were plated into a colony assay; colony-forming unit culture (CFU-C) migration is expressed as the percent of migrated CFU-C of total CFU-C contained in the inoculum (input).

### Actin polymerization assays

BM cells preincubated either with medium or POL5551 (1 μM) were stimulated with 100 ng/ml CXCL12 at 37 °C for the indicated time, fixed in 5% formaldehyde (Carl Roth GmbH, Karlsruhe, Germany) and permeabilized with 0.1% saponin (Carl Roth GmbH), as described.^31^ F-actin was then stained with AlexaFluor568-conjugated phalloidin (Molecular Probes, Eugene, OR, USA) followed by flow cytometric analysis of the relative staining intensity.

### Ca^2+^ flux assay

Ca^2+^ assay was performed with CXCR4-transfected 300-19 murine pre-B cells as described in Supplementary Methods.

### HSPC mobilization

POL5551 (Polyphor Ltd, Allschwil, Switzerland) was suspended in saline and either injected as bolus intraperitoneally (i.p.) or intravenously (i.v.) (0.5–100 μg/g body weight) or filled into continuous-release osmotic minipumps (model 2001, Alzet, Palo Alto, CA, USA), which were implanted under general anesthesia into a dorsal subcutaneous pouch. Mono-biotinylated POL5551 (Polyphor Ltd) was suspended in PBS (Life Technologies GmbH, Darmstadt, Germany) and injected i.p. rhG-CSF (Granocyte, Chugai, Frankfurt, Germany) was suspended in dH_2_0 and diluted in saline to a final concentration of 0.5 μg/μl for i.p. injection. Mice received G-CSF injections every 12 h at a dose of 100 μg/kg for a total of nine doses i.p., referred to as ‘standard regimen' throughout the manuscript. Subsequent blood withdrawal and/or administration of POL5551 were performed directly after the last G-CSF injection on day 5. Cyclophosphamide (CY) or Plerixafor (both from Sigma-Aldrich) were administered as single i.p. injections at doses of 200 mg/kg or 5 and 10 mg/kg, respectively.

### Mouse model of diabetes

Diabetes was induced in 12-week-old C57BL/6 mice with a single i.p. injection of 200 mg/kg Streptozotocin (Calbiochem, Merck Millipore, Darmstadt, Germany) dissolved in citrate buffer (pH 4.7–5.3). Blood glucose levels were measured with a portable glucose meter (Accu-check Aviva, Roche Diagnostics, Mannheim, Germany). Only animals with glucose values higher than 300 mg/dl were used for mobilization experiments 2–3 weeks post Streptozotocin injection.

### Hematopoietic colony assay

For enumeration of CFU-C, aliquots of cells were incubated in duplicate in commercially available growth-factor-supplemented methyl cellulose medium for mouse CFU-C (Stem Cell Technologies, Vancouver, BC, USA or Cell Systems) as described.^23, 32^ CFU-C (BFU-E, CFU-GM and CFU-GEMM) were enumerated after 6–8 days.

### Progenitor cell homing

Progenitor cell homing efficiency was analyzed as described previously.^32^ In brief, lethally irradiated (12.5 Gy) recipients received i.v. transplants of mobilized blood or steady-state BM (ssBM) cells suspended in normal saline. An aliquot of the inoculum was cultured in CFU-C media to quantify the input. Twenty hours after transplantation recipients were humanely killed, and blood, spleen and BM CFU-C contents were enumerated using colony assay. Homing results were evaluated as the fraction of the total injected CFU-C that homed to BM (assuming that 1 femur represents 1/16 of total BM^33^), spleen and blood (assuming 2 ml as total blood volume).

### Engraftment kinetics

Engraftment of different graft sources was tested in a non-competitive setting by transplantation of lethally irradiated (9.5 Gy) mice (CD45.2) with suspensions of mobilized blood cells or BM cells (CD45.1) adjusted to contain ∼1500–2000 CFU-C/recipient based on the data from earlier mobilization studies. Complete blood counts were analyzed every 2–4 days starting on day 12 following transplantation. For complete blood count, 30–40 μl of blood were drawn from the facial vein and analyzed on a hemocytometer (Hemavet 950, Drew Scientific, Dallas, TX, USA).

### Competitive repopulating unit (CRU) assay

To determine the frequency of long-term repopulating HSCs in POL5551 versus G-CSF-mobilized blood, a limiting dilution CRU assay was performed.^34^ Lethally irradiated CD45.2 hosts, 5–10 per group, received i.v. grafts consisting of limiting volumes (2.5, 5.0 and 10 μl) of CD45.1 POL5551 or G-CSF-mobilized blood cells together with 2.5 × 10^5^ CD45.2 BM competitor cells. After 16 weeks, multilineage contribution of the CD45.1 graft-derived leukocytes was measured using flow cytometry. Animals with evidence of mobilized blood-derived (that is, CD45.1+) Gr1+, CD11b+, B220+ and CD3+ cells (⩾0.5% for each lineage) were considered positive for donor cell engraftment. CRU (LTRC) frequency was calculated using the LCALC software (Stem Cell Technologies).

A repopulating unit (RU) assay^35^ was performed to directly compare the repopulating capacity of PB mobilized with different (combinations of) compounds. Lethally irradiated CD45.2 hosts received transplants consisting of a small volumes of blood (CD45.1, 6 μl for POL5551-, Plerixafor- or G-CSF-mobilized blood, 1.5 μl for blood mobilized with G-CSF+POL5551 or G-CSF+Plerixafor) together with 2.5 × 10^5^ CD45.2 BM competitor cells. After 12 weeks, blood graft-derived RUs were calculated for the recipient mice by the following formula: RU=(*D* × *C*)/(100−*D*). *D* is the percentage of blood-derived B and myeloid cells. *C* is the number of RUs cotransplanted with competitor BM (*C*=2.5).

### Tissue processing and immunohistochemistry

Tissue processing and immunohistochemistry were performed as described in Supplementary Methods.

### Detection of biotinylated POL5551

For the detection of biotinylated POL5551, blood collected in heparin-coated tubes (Sarstedt AG & Co, Nümbrecht, Germany) was treated directly with the crosslinking reagent Bis[sulfosuccinimidyl] suberate (BS^3^, Thermo Fisher Scientific Inc, Rockford, IL, USA) at a final concentration of 5–10 mM (at first resuspended in PBS, Life Technologies GmbH). BM was flushed in PBS and resuspended in fresh 5 mM BS^3^ solution. Crosslinking was performed at room temperature for 30 min followed by quenching of the reaction with 15 mM Tris-HCl (pH 7.5, Carl Roth GmbH). Subsequent fixation of the samples was carried out with 5% formaldehyde (Carl Roth GmbH), followed by staining with streptavidin and anti-CD45 antibody performed simultaneously in fresh PBS/bovine serum albumin.

### Human subjects' protection

Human cells, which served as the source for CXCR4 mRNA, were from anonymized leftover materials from quality control samples, used with permission of the local internal review board (IRB, no. 329/10) in agreement with the WMA Declaration of Helsinki. Written donor approval was obtained.

### Statistics

Descriptive statistics and Student's *t*-tests, with Bonferroni correction where indicated, were calculated using Excel (Microsoft, Redmond, WA, USA).

## Results

### POL5551 is a potent CXCR4 antagonist

**Figure 1:**
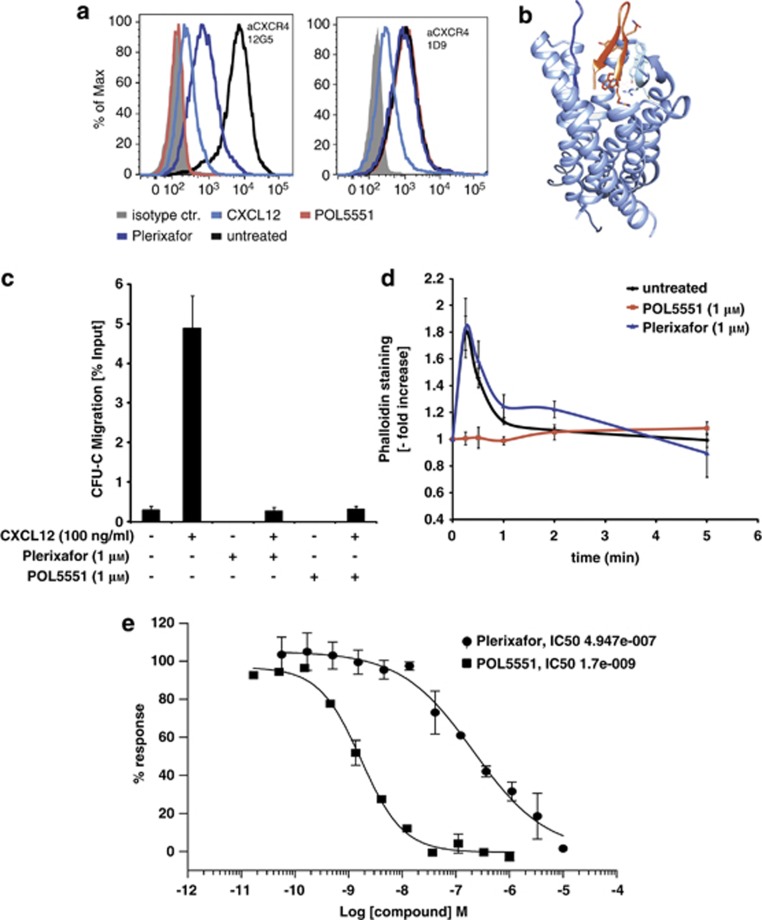
POL5551 is a CXCR4 antagonist. (**a**, **b**) Analysis of the binding properties of POL5551 to CXCR4. (**a**) Ao.o1 cells overexpressing human CXCR4 were incubated with CXCL12, Plerixafor or POL5551 (1 μM for all) plus anti-CXCR4 antibody (Ab) clones 12G5 (extracellular loops) or 1D9 (N-terminus). CXCR4 Ab without agonist/antagonists (untreated) or isotypic control Ab (isotype) were used as positive and negative controls. (**b**) Structural model demonstrating the interaction of hairpin-shaped peptide POL5551 (in red) with the extracellular loops of CXCR4 (PDB file 3OE0). (**c**, **d**) Effects of POL5551 on *in vitro*migration and polymerization of F-actin filaments: BM cells were incubated with PBS/bovine serum albumin (BSA) or Plerixafor or POL5551 (1 μM in PBS/BSA) and then subjected to transwell migration for 4 h ((**c**) mean±s.e.m., *n*=3 for Plerixafor- or POL5551-treated samples, *n*=10 and 13 for spontaneous migration (medium only) and migration towards CXCL12 (100 ng/ml), respectively) or stimulation with CXCL12 (100 ng/ml) with subsequent Phalloidin staining (**d**) mean±s.e.m., *n*=3 for Plerixafor- or POL5551-treated samples, *n*=6 for untreated BM. (**e**) Determination of POL5551 IC50 value: calcium flux response to CXCL12 stimulation. CXCR4-transfected murine pre-B cells (300-19) were treated with different concentrations of POL5551 or Plerixafor and stimulated with CXCL12. The resulting percentage of inhibition of CXCL12-induced Calcium flux was used to calculate the IC50 value (*n*=20). Representative inhibition curves from duplicate measurements are shown.

We compared binding properties of POL5551 to those of the natural ligand, the chemokine CXCL12,^36^ and the well-characterized CXCR4 antagonist Plerixafor.^37^ Binding of two CXCR4 antibodies (Abs), clone 12G5 (which binds extracellular loops 1 and 2^27^) and clone 1D9 (which recognizes an epitope within the N-terminus^27^), was tested using flow cytometry after concurrent incubation of Ao.o1_hCXCR4 cells with Abs and compounds (Figure 1a and Supplementary Figure S1). In agreement with previous reports, Plerixafor interfered with 12G5 binding without affecting the binding of 1D9.^27, 38^ By contrast, CXCL12 blocked the binding of both clones, indicative of its interaction with both the extracellular loops and the N-terminus, again in agreement with published data.^38^ Similar to Plerixafor, POL5551 bound to the extracellular loops but not to the N-terminal moiety recognized by 1D9. This was also confirmed by the molecular model of a POL5551 analog^39^ bound to CXCR4 (Figure 1b).

We next sought to confirm antagonistic properties of POL5551 in functional *in vitro* assays. Responsiveness of cells pretreated with either Plerixafor or POL5551 (both at 1 μM) to CXCL12 was assessed by standard chemotaxis and F-actin polymerization assays. CXCL12-induced transwell migration of ssBM CFU-C (∼6%) was completely blocked by pre-incubation with either of the CXCR4 inhibitors (Figure 1c). By contrast, whereas POL5551 pretreatment completely abrogated polymerization of F-actin filaments following CXCL12 stimulation, Plerixafor did not show an inhibitory effect in this assay (Figure 1d). A quantitative comparison of POL5551- and Plerixafor-mediated inhibition of cellular Ca^2+^-Flux was performed (Figure 1e). The resulting value of 2–3 nM for POL5551 was ∼200-fold lower than the IC_50_ concentration determined for Plerixafor (400–600 nM). Thus, except for the chemotaxis assay, which favors slowly acting antagonists because of the long incubation time (4 h) and where the activity of the antagonists was the same, *in vitro* performance of POL5551 as the CXCR4 inhibitor was superior to that of Plerixafor.

### Rapid and potent mobilization of hematopoietic progenitor cells by POL5551

**Figure 2:**
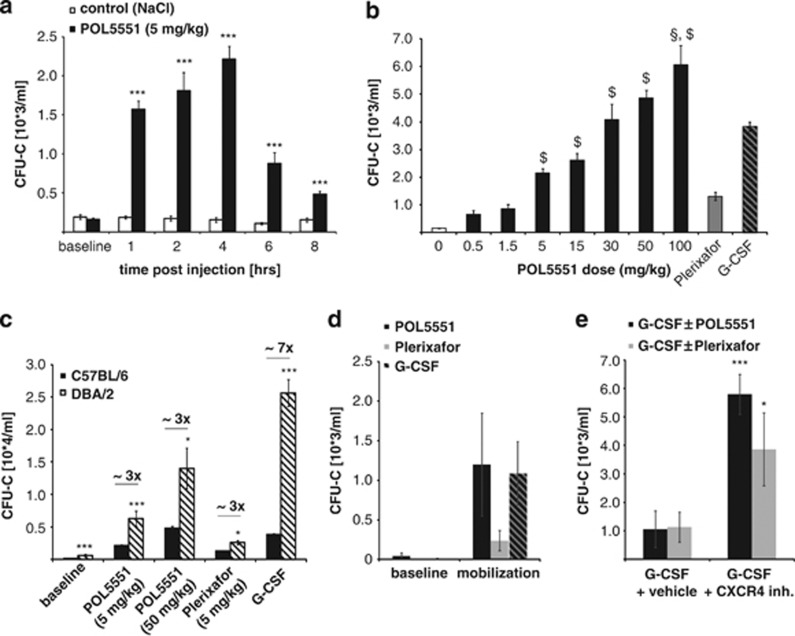
Mobilization of murine hematopoietic stem and progenitor cells with POL5551. (**a**) Time response kinetics: C57BL/6 mice received POL5551 (5 mg/kg) or NaCl (control) i.p. and blood was drawn at the indicated time points for CFU-C enumeration (mean±s.e.m. from 10–12 mice per time point for POL5551 and 6–9 mice per time point for control mice; two independent experiments). (**b**) Dose response to escalating doses of POL5551: POL5551 was injected i.p. at indicated doses and blood was drawn 4 h later (*n*=6 mice for all groups except 5 mg/kg, *n*=15 mice). Control mice received saline (*n*=14), a standard regimen of G-CSF (*n*=58) or a single injection of Plerixafor (5 mg/kg, i.p., *n*=4). Significant (*P*<0.05) superiority to G-CSF (§) or Plerixafor ($) is indicated above the bars (mean±s.e.m.). (**c**) Mouse strain-related potency of POL5551: DBA/2 mice received mobilizing agents as indicated (standard regimen of G-CSF was administered); blood was drawn for CFU-C enumeration at optimal time points as described above (mean±s.e.m., *n*=8 per condition from two independent experiments). Values from C57BL/6 mice are shown for comparison. (**d, e**) Mobilization in diabetic mice: SZT-treated C57BL/6 mice were analyzed for PB CFU-C counts at baseline as well as following mobilization with POL5551 (30 mg/kg, i.p. 4 h after injection, mean±s.e.m., *n*=3), Plerixafor (10 mg/kg, i.p., 1 h after injection, mean±s.e.m., *n*=3) or G-CSF (standard regimen, mean±s.e.m., *n*=6) (**c**). (**d**) G-CSF-treated mice were subsequently treated with Plerixafor (10 mg/kg, i.p., mean±s.e.m., *n*=3) or POL5551 (30 mg/kg, i.p., mean±s.e.m., *n*=3) following the ninth G-CSF dose. Mobilized CFU-C were quantified 1 (Plerixafor) or 4 h (POL5551) thereafter. ***P<0.001, *P<0.05.

Time and dose responsiveness of HSPC egress after POL5551 injection were evaluated next in C57BL/6 mice. CFU-C mobilization after a single bolus injection of POL5551 (5 mg/kg, i.p., Figure 2a) occurred rapidly with a significant increase observed at 1 h (1500 CFU-C/ml) and a peak reached after 4 h (2200 CFU-C/ml), representing a 10- and 14-fold increase, respectively, compared with baseline circulating CFU-C levels (∼160 CFU-C/ml). The majority of mobilized progenitors disappeared from the circulation quickly thereafter. Peak plasma concentration of the compound was reached 1 h after injection; after 4 h, >90% of POL5551 had been cleared from the circulation (Supplementary Figure S2A). After i.v. administration of POL5551, mobilization kinetics were similar to the i.p. treatment, whereas the efficiency was increased by >50%. (Supplementary Figure S2B).

Whole blood count analysis showed a peak of white blood cell mobilization at 2 h after POL5551 injection (Supplementary Figure S3A). Compared with control groups receiving G-CSF (standard regimen) or bolus injection of Plerixafor (5 mg/kg, i.p.), no significant differences were found in the relative composition of mature leukocyte subsets in POL5551-mobilized blood. The frequency of neutrophils was increased in mobilized (most prominently in G-CSF-mobilized) versus non mobilized blood (Supplementary Figure S3B). Further analysis of mobilized subsets confirmed the relative increase in the myeloid fraction (Gr1+, Mac1+) in mobilized blood (Supplementary Figure S3C). No differences in the ratio of cytotoxic T cells and T-Helper cells were observed (Supplementary Figure S3D).

Injection of escalating doses of POL5551 (0.5–100 mg/kg, Figure 2b and Supplementary Figure S4) resulted in a positive, non-linear dose response of mobilized CFU-C for the doses tested. Mobilization achieved with Plerixafor (5 mg/kg, i.p.) or the standard regimen of G-CSF was in the range of what has been reported previously by us and others.^16, 23, 40^ At doses >5 mg/kg, POL5551 induced HSPC mobilization (2200 CFU-C/ml) to significantly higher levels than Plerixafor (1300 CFU-C/ml). Moreover, at doses of 20–30 mg/kg, mobilization levels (2800–4000 CFU-C/ml) were comparable to and at higher doses even exceeded mobilization with G-CSF (3800 CFU-C/ml, Figure 2b, and data not shown).

To assess the magnitude of the difference in mobilization of C57BL/6 and DBA/2 mice in response to POL5551, we next evaluated responsiveness of DBA/2 mice to POL5551 (5 and 50 mg/kg, i.p.), with G-CSF- and Plerixafor-treated mice as controls. Indeed, at both doses mobilization with POL5551 was increased by almost threefold in DBA/2 relative to C57BL/6 mice (Figure 2c) similar to the relative increase found with Plerixafor between the two strains. G-CSF mobilized at least six times more CFU-C in DBA/2 than in C57BL/6 mice.

Streptozotocin-induced diabetic mice (type 1 diabetes^41^) were used as a disease model of G-CSF refractoriness. POL5551 (30 mg/kg)-treated mice were compared with mice treated with G-CSF (standard regimen) and Plerixafor (10 mg/kg, i.p.). Both CXCR4 antagonists were therefore tested at equimolar doses. Treatment with all three agents resulted in markedly decreased (to approximately one-fourth) mobilization in diabetic as compared with healthy mice (Figures 2b and d). Addition of either of the CXCR4 inhibitors (30 mg/kg for POL5551 and 10 mg/kg for Plerixafor) after the ninth G-CSF dose could rescue diabetes-induced hyporesponsiveness to G-CSF (Figure 2e).

### POL5551 synergizes with G-CSF and CY

**Figure 3:**
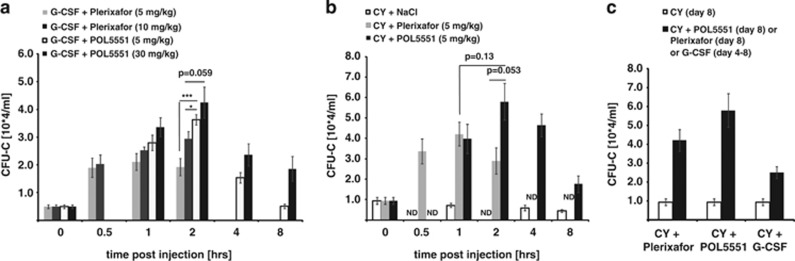
Synergism of POL5551 with other mobilizing modalities. (**a**) Co-mobilization with G-CSF and POL5551 or Plerixafor: after a standard regimen of G-CSF treatment (day 5), mice received a single i.p. injection of POL5551 (5 or 30 mg/kg) or Plerixafor (5 or 10 mg/kg). Circulating CFU-Cs were enumerated at the indicated time points (mean±s.e.m., *n*=6–8 mice). (**b**) Kinetics of mobilization with Cyclophosphamide (CY) and POL5551 or Plerixafor: mice received a single dose of CY (200 mg/kg, i.p.). Circulating CFU-C numbers were enumerated on day 8 after CY injection, immediately before (mean±s.e.m., *n*=14) as well as 1, 2, 4 and 8 h after POL5551 (5 mg/kg, i.p., mean±s.e.m., *n*=8–18) or saline (mean±s.e.m., *n*=8–11) or 0.5, 1 and 2 h after Plerixafor (5 mg/kg, i.p., *n*=5–15) injection. (**c**) Combination of CY and G-CSF or POL5551: mice received CY (200 mg/kg, i.p.) on day 0 plus standard regimen of G-CSF on days 4–8 (mean±s.e.m., *n*=8). For comparison, the circulating CFU-C numbers from mice treated with CY only on day 8 as well as from mice that received POL5551 (5 mg/kg, 2 h) or Plerixafor (5 mg/kg, 1 h) on day 8 from **b** are shown. ***P<0.001, *P<0.05, ND: not determined.

Synergistic mobilization by Plerixafor and G-CSF has been reported for various treatment schedules of both agents.^21, 23^ We therefore tested whether a POL5551 bolus injection (5 or 30 mg/kg, i.p.) given at the end of a standard regimen of G-CSF could similarly enhance mobilization. Mice mobilized with the combination of G-CSF and Plerixafor (5 or 10 mg/kg) served as controls, with 10 mg/kg of Plerixafor and 30 mg/kg of POL5551 representing equimolar doses of the inhibitors. In the combined treatment regimens, mobilization was noticeably enhanced (Figure 3a).

The combination of POL5551 or Plerixafor with the cytotoxic agent CY was investigated next. On day 8 after CY injection, when peak mobilization occurs,^42^ addition of a single dose of POL5551 or Plerixafor (both at 5 mg/kg, i.p.) mobilized >50 000 or >40 000 CFU-C per ml PB, respectively (Figure 3b). The synergism between CY and POL5551 or Plerixafor was more pronounced than the combination of CY plus G-CSF (Figure 3c).

### Properties of POL5551-mobilized stem and -progenitor cells

**Figure 4:**
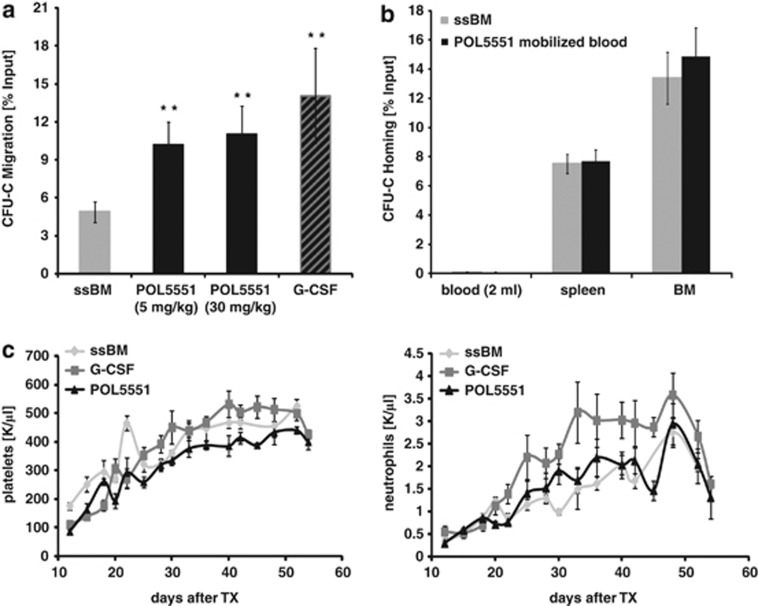
Properties of POL5551-mobilized HSPCs. (**a**) CXCL12 responsiveness of POL5551-mobilized HSPCs: mice received a single injection of POL5551 at the indicated dose or standard regimen of G-CSF. Migration of PB-mobilized CFU-C towards CXCL12 was assessed by a transwell migration assay and compared with migration of steady-state BM (mean±s.e.m., *n*=5–8 for mobilized blood specimen, POL5551-mobilized blood was drawn 4 h after the injection, *n*=13 for steady-state BM). (**b**) Homing of POL5551-mobilized HSPCs: Lethally irradiated (12.5 Gy) recipients received injections estimated to contain ∼10 000 CFU-C from POL5551-mobilized blood (continuous infusion, 30 mg/kg/day) or steady-state BM cells. An aliquot of the inoculum was cultured in CFU-C media to quantify the input. After 20 h, CFU-C content in blood, spleen and BM of recipient mice was similarly analyzed. Homing is expressed as the ratio of the number of CFU-C recovered from each of the three organs over the total number of injected CFU-C (mean±s.e.m., *n*=13–15 from three independently performed experiments). (**c**) Engraftment kinetics of POL5551-mobilized HSPCs: radiation-conditioned (9.5 Gy) recipients received a graft of POL5551-mobilized blood (*n*=6 donor mice) or (control groups) G-CSF-mobilized blood (*n*=6 donor mice) or steady-state BM cells (*n*=2–3 donor mice). Reconstitution of hematopoiesis was assessed using blood count analysis for the indicated timespan. The graphs showing the kinetics of platelet (left) and neutrophil (right) engraftment are representative of two experiments (mean±s.e.m., *n*=8–10 per recipient group). **P<0.01.

If POL5551 mobilizes HSPCs by directly targeting the CXCR4 receptor, this raises the question whether as a consequence POL5551-mobilized cells found in circulation can still sense CXCL12. We therefore performed migration assays with POL5551-mobilized blood HSPCs (Figure 4a). At both doses tested (5 and 30 mg/kg), POL5551-mobilized CFU-Cs were highly responsive towards the chemokine signal, more so than untreated BM and to a similar degree as was also observed for G-CSF-mobilized blood. All three mobilized specimen had lower expression of cell adhesion receptors when compared with ssBM progenitors (Supplementary Table S2). Interestingly, CXCR4 surface expression on POL5551-mobilized progenitors was significantly higher relative to ssPB (Supplementary Figure S5).

There is controversy regarding the role of the CXCR4/CXCL12 pathway for efficient homing of HSPCs.^25, 43, 44, 45^ Given the unprecedented potency of POL5551 as CXCR4 antagonist, we tested how efficiently POL5551 mobilized CFU-C home to the BM of lethally irradiated recipients by determining the recovery of donor cells from hematopoietic organs 20 h after transplantation. As shown in Figure 4b, homing of POL5551-mobilized CFU-C was as efficient as that of ssBM. Our next experiment consequently addressed whether POL5551-mobilized progenitors can also provide timely early engraftment. As determined by serial blood count analyses, all three examined transplant sources, ssBM, as well as G-CSF- or POL5551-mobilized blood, showed similar kinetics of engraftment in lethally irradiated hosts (Figure 4c).

### POL5551 mobilizes CRU

**Figure 5:**
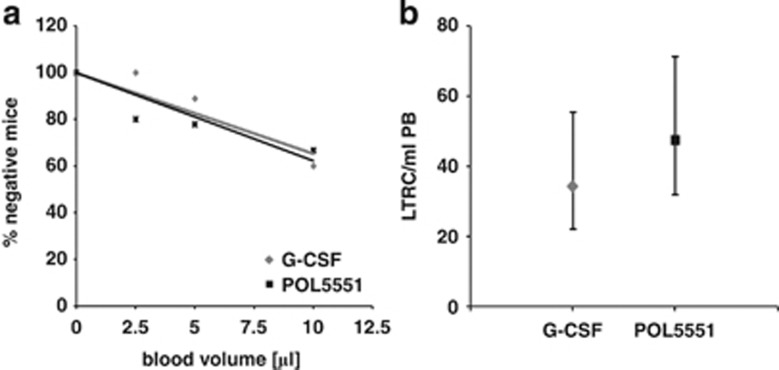
Mobilization of CRU by POL5551. CRU frequency in POL5551 (30 mg/kg)-mobilized blood was determined using a CRU assay and compared with the CRU frequency in G-CSF (standard regimen)-mobilized blood. Lethally irradiated recipients (*n*=5–10 per group) received transplants of 250 000 BM competitor cells (CD45.2) together with indicated limiting volumes of mobilized blood (CD45.1, 3 pooled donors per experimental group). CRU engraftment, defined as multilineage engraftment of ⩾0.5% per lineage was quantified 16 weeks after transplantation. (**a**) Percentages of negative mice were plotted against blood volume; f(x)=−3.4709*x*+100 (*R*^2^=0.86) for G-CSF and f(*x*)=−3.7672*x*+100 for POL5551 (*R*^2^=0.75). The mean CRU (LTRC) frequency (**b**) was calculated using Poisson's statistic (LCALC software, Stem Cell Technologies) (mean±upper/lower frequency).

The frequency of long-term RUs in POL5551 bolus-mobilized blood was quantified and compared with G-CSF-mobilized blood using a standard limiting dilution CRU assay.^34^ Based on the dose response studies depicted in Figure 2b, we selected a dose of POL5551 (30 mg/kg, i.p.) that induced CFU-C mobilization in the range of G-CSF. The proportion of engrafted mice increased with the volume of transplanted blood (Figure 5a). At the doses used, POL5551 and G-CSF mobilized CRU into blood at similar frequencies (47 and 34 CRU/ml blood, respectively, Figure 5b).

In addition, a RU assay was performed to directly compare the RU concentration in blood from mice mobilized with G-CSF, Plerixafor (10 mg/kg, i.p.) or POL5551 (30 mg/kg, i.p.), as well as with G-CSF in combination with Plerixafor or POL5551. The relative concentration of RU in each of the mobilized specimen replicated the agents' (agent combinations') efficiency at CFU-C mobilization (Supplementary Figure S6).

### POL5551 treatment has minimal effects on macrophage and osteoblast distribution within the BM and endosteal microenvironments

**Figure 6:**
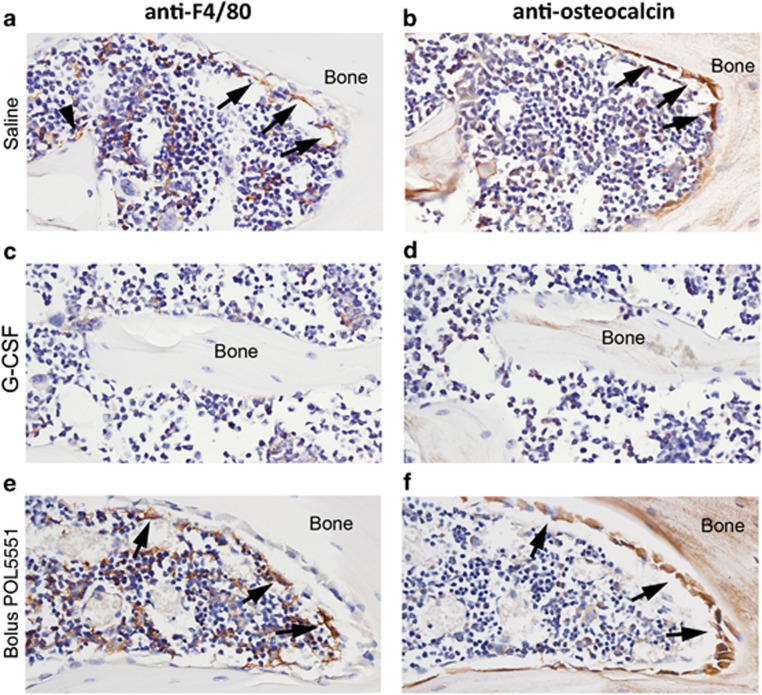
Macrophage and osteoblast distribution within the BM in response to mobilizing agents. Immunohistochemical staining of bone and BM collected from mice treated with saline (**a**, **b**), G-CSF (**c**, **d**), bolus POL5551 delivery (**e**, **f**). Specific antibody staining (brown) was performed using antibodies for F4/80 (left panel) or osteocalcin (right panel) and confirmed by comparison to isotype-matched control staining within the same experiment (data not shown). All sections were counterstained with hematoxylin (blue nuclei). Images within treatment groups are from serial sections. Bone matrix is demarked as ‘Bone' and this text is placed in a similar location in paired images, providing a landmark reference point. Arrows indicate canopy F4/80+ osteomacs in the left panel or mature osteocalcin+ osteoblasts in the right panel. Arrowhead in **a** indicates a resting bone surface F4/80+osteomac. Original magnification of all images is × 40.

We previously confirmed that mobilization of HSPC using either G-CSF or CY, but not Plerixafor, occurs through a mechanism that initiates collapse of HSC niche cellular components.^17^ Using a similar immunohistochemistry approach, we examined whether the distribution of mature osteoblasts and macrophages within the BM and endosteal environment was disturbed by treatment with POL5551. Observations are reported relative to saline-treated control samples that exhibited expected cellular distributions for skeletally mature mice (Figures 6a and b). In contrast to G-CSF (Figures 6c and d), treatment with POL5551 had no apparent effect on macrophage or osteoblast distribution within the BM and endosteum. The F4/80+osteomac canopy (Figure 6e, arrows) covering osteocalcin+ osteoblasts (Figure 6f, arrows) within the endosteal region was clearly maintained. Similarly, the distribution and relative number of F4/80+ macrophages within the BM (Figure 6e) were indistinguishable from saline-treated mice.

### POL5551 mobilizes hematopoietic and progenitor cells by targeting them directly

**Figure 7:**
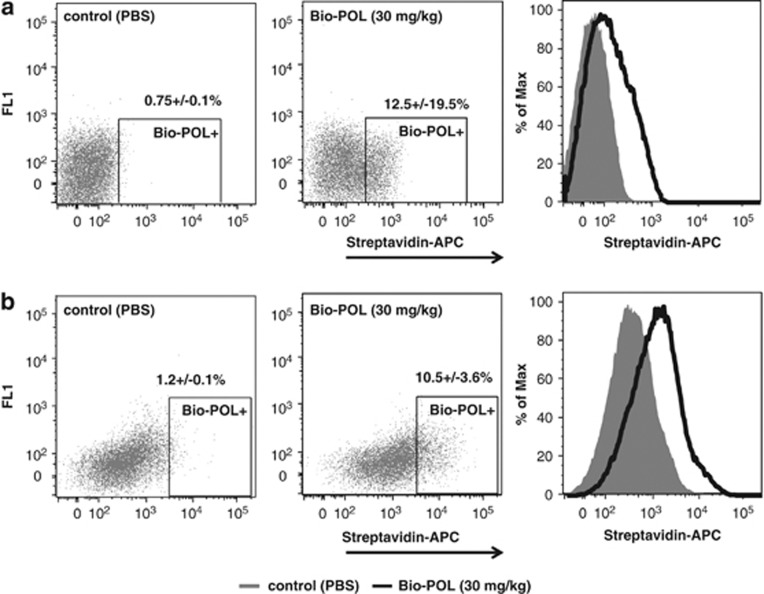
Targeting of hematopoietic cells by POL5551. POL5551 labeled with a single biotin molecule (Bio-POL) was injected i.p. (30 mg/kg). Control mice received PBS. Thirty minutes after the injection, blood and BM CD45+ cells were analyzed for the presence of Bio-POL on their surface using fluorescence-coupled streptavidin. **a** and **b** show representative stainings of blood and BM samples, respectively (mean±s.e.m., *n*=3, 3 independent experiments).

By injecting biotin-labeled POL5551 (Bio-POL) into mice and analyzing BM and blood for Bio-POL binding at time points preceding mobilization (30 min after injection), we sought to determine whether it directly targets HSPCs. Indeed, Bio-POL was detected on hematopoietic cells (CD45-positive) in the BM within 30 min of i.p. injection (Figure 7a), as well as on circulating cells (Figure 7b). In addition, POL5551 was detected in BM fluid samples prepared from treated animals (Supplementary Figure S7).

## Discussion

In this study, *in vitro* and *in vivo* properties of the novel CXCR4 antagonist POL5551 as a mobilizing agent were evaluated. The markedly improved potency of POL5551 compared with Plerixafor was shown by its superior ability to block CXCL12-induced responses *in vitro* and reflected in its *in vivo* efficacy. Dose escalation of POL5551 as a single mobilizing agent resulted in mobilization in excess of G-CSF-induced mobilization, which to our knowledge was not previously achieved by CXCR4 antagonists in mice. Of note, mobilization with Plerixafor in mice was found to peak at 5 mg/kg^21, 23^ and could not be tested at doses >10 mg/kg because of its toxicity, whereas POL5551 was well tolerated up to a dose of 100 mg/kg. Contrary to what we previously proposed,^23^ these data indicate that the restricted efficacy of Plerixafor in mice might not be because of the limited size of CXCR4-antagonist-sensitive HSPC pools, but rather to its limited potency. Furthermore, in the model of diabetes-induced G-CSF refractoriness, POL5551 was more effective in mobilizing progenitors than Plerixafor.

Differences in mobilization response of various mouse inbred strains—best studied for G-CSF but also noted for other mobilizing agents—are well documented^46, 47^ and thought to correspond to the variable response to G-CSF in humans. By comparison, C57BL/6 mice are relatively poor mobilizers, whereas DBA/2 mice respond with considerably stronger mobilization to various stimuli.^21, 47^ The rapid mobilization kinetics of Plerixafor and POL5551 would not allow for prior HSPC proliferation when given as single injection. The differences between C57BL/6 and DBA/2 mice in mobilization response to these CXCR4 inhibitors therefore suggest strain-specific differences in mice (presumably modelling individual-specific differences in humans) in the firmness of CXCL12-mediated stem cell retention or in the relative microanatomical distribution of the cells within the BM.

Synergistic mobilization by G-CSF and CXCR4 antagonists has generally been attributed to only partial targeting of the CXCR4/CXCL12 pathway as well as expansion/relocation of CXCR4 antagonist-mobilizable pools over the course of G-CSF treatment.^1, 23^ In agreement with previous reports, G-CSF-mediated mobilization was markedly enhanced by the addition of POL5551. Moreover, the combination of POL5551 or Plerixafor with the cytotoxic agent CY resulted in synergistic mobilization in excess of that observed with G-CSF plus CY, which can be explained by the significant overlap in pathways targeted by G-CSF and CY.^7, 48^

The RU assay confirmed the relative potency of single agents as well as the combination of G-CSF with CXCR4 antagonists, reproducing the CFU-C mobilization pattern associated with these modalities. Limiting dilution competitive transplantation assays with POL5551-mobilized blood demonstrated the presence of CRUs, as experimental evidence of mobilization of true stem cells. The evidence provided is critically important if clinical transplantation of POL5551-mobilized stem cell grafts is planned.

The cellular integrity of the endosteal region was maintained after treatment with POL5551. These data demonstrating differential effects on marrow architecture by G-CSF and POL5551 are not unexpected. The rapid kinetics of CXCR4 antagonists would likely not allow for significant architectural changes in the BM, and indeed similar data were previously reported with the CXCR4 antagonist Plerixafor.^17^ Nevertheless, given the several-fold weaker action of Plerixafor, the absence of BM remodeling in response to POL5551 was not self-evident.

Brisk responsiveness of POL5551-mobilized HSPCs to CXCL12 *in vitro* is in agreement with reported data on efficient CXCL12-directed transwell migration as a common property of all mobilized specimen.^32^ The observed efficient homing of POL5551-mobilized progenitors is consistent with publications about the homing of CXCR4-deficient or of Plerixafor-mobilized cells.^21, 23, 25^

It has been assumed that the molecular mechanism of mobilization of HSPCs by CXCR4 antagonists *in vivo* is disruption of the CXCL12/CXCR4 axis at the level of the HSPC, and consequently, egress of HSPCs deprived of CXCL12 signaling input derived from the marrow stroma. However, CXCR4 antagonist-mobilized HSPCs do respond to CXCL12 *in vitro* (see above and also refer to other studies^21, 23, 27^). As a result, it has been recently hypothesized that CXCR4 antagonists also target the stroma cells, causing an alteration of the CXCL12 gradient and eliciting HSPC egress by this indirect mechanism.^28^ Proof of either of the hypotheses hinges on the demonstration of the antagonist binding to CXCR4 on HSPCs in the BM at early time points after injection of the compound—that is, prior to mobilization. Detection of biotin-labeled POL5551 on the surface of hematopoietic cells in BM of Bio-POL-mobilized animals demonstrated here is the first direct evidence of binding of CXCR4 antagonists to HSPCs *in vivo* supporting direct targeting of HSPCs by CXCR4 antagonists as the mechanism underlying their mobilization. Whether attenuation of the CXCL12 gradient between the BM and plasma also contributes to cell egress cannot be excluded.

## Concluding remarks

In summary, we demonstrate that POL5551 is a fast-acting, efficient and safe mobilizing agent for immature hematopoietic cells, including long-term repopulating stem cells. At higher doses, its potency exceeds that of G-CSF in C57BL/6 mice, which sheds new light on the size of CXCR4 antagonist-mobilizable pools. With respect to mechanisms of mobilization by CXCR4 inhibitors, we provide evidence supporting the notion that mobilization with POL5551 is the result of direct targeting of CXCR4 on HSPCs in the BM. Provided that the data can be corroborated in humans, POL5551 possesses promising therapeutic potential alone or in combination with the standard mobilizing agents.

## Notes

BR, KP, EC and KD are employees of Polyphor that provided the compound and partially funded the studies described. The remaining authors declare no conflict of interest.
