# Supplementary material for: The novel CXCR4 antagonist POL5551 mobilizes hematopoietic stem and progenitor cells with greater efficiency than Plerixafor
Source: Leukemia. 2013 Sep 27;27(12):2322–31. doi: 10.1038/leu.2013.266 (PMC3865534; doi:10.1038/leu.2013.266)
Supplement: Supplementary Information [file leu2013266x1.doc]

**Supplemental Methods**

**Cloning of human CXCR4**

mRNA was isolated from primary human peripheral blood progenitor cells and transcribed into cDNA (Promega Corporation, Madison, WI). cDNA of human CXCR4 (hCXCR4) was amplified using specific primer sequences with BglII or XhoI restriction sites, respectively (5’-gatcagatctgccaccatgtccattcctttgc -3’ (fwd) and 5’-gatcctcgagccacgggaatggagagatta-3’ (rev)). The cDNA was cloned into the retroviral vector pRetroX-IRES-DsRedExpress (Clontech Laboratories, Inc. Mountain View, CA) in exchange for the reporter gene DsRed. Transduction of Ao.o1 cells was performed using the pantropic retroviral expression system (Clontech Laboratories, Inc. Mountain View, CA). Briefly, GP2-293 cells were plated in 6-well plates (2.5 x 10^5^ cells per well in DMEM, 10% FCS, Pen/Strep, Glutamate). On day 1 cells were cotransfected with the envelope encoding vector pVSV-G (Clontech Laboratories, Inc. Mountain View, CA) and the CXCR4 encoding vector using Lipofectamine (Life Technologies, Carlsbad, CA) according to manufacturer’s instructions. Media were changed within 12 hours of transfection. After 48 hours supernatant was collected, filtered through a 0.22 µm filter and added to freshly prepared Ao.o1 cells (1:1 fresh RPMI medium + GP2-293 supernatant). After additional 48 hours the media were exchanged. Analysis of cells for CXCR4 expression by FACS was performed three days later followed by two sorting rounds of hCXCR4 positive cells up to a purity of ≥ 96%.

**Ca^2+^ Flux-Assay**

CXCR4 transfected 300-19 murine pre-B cells were labeled as a batch with Calcium4 Reagent (Molecular Devices, Downingtown, PA) in HBSS + 20 mM Hepes + 0.1%BSA buffer for 40 min at 37 °C. After dispensing 8 x 10^4^ cells in each well of black 384-well plates, the plate was centrifuged and placed in a FlipR384 (Molecular Devices, Downingtown, PA) automated plate reader. After reading a 20 s baseline, the FlipR384 dispensed diluted PEM compounds to the plate. Signal was recorded for 200 s before addition of CXCL12 (Peprotech, Rocky Hill, NJ or CellSystems) at a final concentration of 10 nM in HBSS + 20mM Hepes + 0.1% BSA onto the cells. Calcium flux was measured for an additional 200 s. The maximum and minimum signals were determined from control wells without inhibitor (POL5551 or Plerixafor) or without CXCL12, respectively. Percentage of inhibition was calculated from a range of compound concentrations, which were subsequently applied to calculate IC50 values using GraphPad Prism software (GraphPad Software Inc., La Jolla, CA). All steps in FlipR384 were carried out at room temperature.

**Pharmacokinetics**

**Plasma** preparation: blood samples were collected in tubes containing very small amounts of heparin (15 µl). **BM fluid** preparation: freshly isolated femurs and tibias were flushed in minimal volume (300-500 µl) of cold PBS. If not processed immediately fresh samples/bones were stored on ice. After centrifugation (15-20 min, 3000-4000 rpm, 4°C) plasma/BM fluid supernatant was carefully removed, frozen and stored at <-20 °C until just before analysis. Analysis: Concentrations of POL5551 in plasma and bone marrow were determined using high pressure liquid chromatography coupled to mass spectrometry detection (LC‑MS/MS analytical method). Briefly, after addition of an internal standard (POL6326), plasma samples (aliquot of 50 µL) and bone marrow fluid samples (aliquot of 20 µL) were extracted with acetonitrile (acidified with formic acid). Supernatants were evaporated to dryness under a stream of nitrogen, and reconstituted in H_2_O/ ACN, 95/5, v/v, +0.2% formic acid. Extracts were then analyzed by reverse-phase chromatography (Acquity BEH C18 column, 100 x 2.1 mm, 1.7 µm column), using an acidified water /acetonitrile gradient elution (UPLC, Waters). The detection and quantification was performed by mass spectrometry, with electrospray interface in positive mode and selective fragmentation of analytes (AB Sciex 4000 Q Trap mass spectrometer). Standards, Quality Controls and samples were extracted and assayed in the same manner.

**Tissue processing and immunohistochemistry**

Dissected hind limbs were fixed for 24 hrs in 4% paraformaldehyde (Sigma, St Louis, MO, USA) at 4 °C. Bones were subsequently decalcified using 14% ethylenediaminetetraacetic acid (Sigma, St Louis, MO, USA) pH 7.2 at 4 °C for a minimum of 2 weeks. All specimens were processed and paraffin embedded using a Shandon Pathcenter Processor and embedding station using extended processing times suitable for hard tissue embedding (Thermo Electron Corporation, Waltham, MA, USA). Immunohistochemistry (IHC) was performed as described elsewhere(1). Tissue staining was viewed and captured using a Nikon eclipse 80i microscope with a Nikon D5-Ri1 camera and NIS-elements imaging software. Qualitative assessment of samples was performed blinded with representative images collected within similar areas of the metaphyseal region (original magnification 40x). Digital editing was performed using Adobe Photoshop with minor modifications made to the entire image to reduce capture artifacts.

**Modeling**

From the average NMR structure bundle of POL3026 (an analogue of POL5551 and the bicyclic analogues of the cyclic peptide CVX15 bound to CXCR4(2)) one typical structure was selected. The model was built by superimposition of backbone atoms in the 10-membered ring of the NMR structure with the corresponding region of the cyclic peptide bound to CXCR4 (PDB: 3OE0). Both ring structures contain the D-Pro-L-Pro template and adopt regular ß-hairpin conformations.

**Data analysis**

Mean values of CFU-C mobilized per ml peripheral blood as a function of different doses tested were subjected to multiple (linear and non-linear) regression analysis using CurveExpert software (Hyams, D. G., CurveExpert 1.4, Chadwick Court Hixson, TN). The Morgan-Mercer-Flodin (MMF) regression model (f(x)=(ab+cx^d)/(b+x^d), estimated parameters: a=1.42, b=1.83, c=1.4, d=5.7) was determined as best fitting curve (correlation coefficient: R^2^=0.99) indicating a non-linear (sigmoid) relationship between the increase in the numbers of circulating progenitors and POL5551 dose.

**Antibodies**

Antibodies used in this study are listed in Table S1.

**Table S**1: Antibodies

| Antibody | Clone | Conjugate | Source |
| --- | --- | --- | --- |
| CXCR4 (human) | 12G5 | PE | BD |
| CXCR4 (human) | 1D9 | PE | BD |
| CXCR4 (murine) | 2B11 | PerCP-eFluor®710 | eBioscience |
| F-Actin | Phalloidin | AlexaFluor488 | Molecular Probes |
| CD45.1 (mouse) | A20 | PE | BD |
| CD45.2 (mouse) | 104 | FITC | BD |
| CD45.2 (mouse) | 104 | eFluor®450 | eBioscience |
| CD45 (mouse) | 30-F11 | eFluor®450 | eBioscience |
| CD45 (mouse) | 30-F11 | APC | BD |
| Gr-1/Ly-6G and C (mouse) | RB6-8C5 | Biotin | eBioscience |
| Gr-1/Ly-6G and C (mouse) | RB6-8C5 | APC-Cy7 | BioLegend |
| CD11b (Mac1) | M1/70 | FITC | eBioscience |
| CD11b (Mac1) | M1/70 | PE | eBioscience |
| CD45R (B220) | RA3-6B2 | PE-Cy7 | eBioscience |
| CD3 | 17A2 | Alexa Fluor ® 647 | BD |
| CD3 | 17A2 | eFluor®450 | eBioscience |
| CD4 | GK1.5 | APC | eBioscience |
| CD8 | 53-6.7 | PerCP-Cy5.5 | eBioscience |
| CD117 (c-kit) | 2B8 | APC | BD |
| CD117 (c-kit) | ACK2 | PE-Cy7 | eBioscience |
| CD49d | R1-2 | PE | BD |
| CD49e | 5H10-27 (MFR5) | PE | BD |
| Antibody | Clone | Conjugate | Source |
| CD49f | GoH3 | PE | BD |
| CD29 | Ha2/5 | FITC | BD |
| CD26 | H194-112 | FITC | BD |
| VCAM1 (CD106) | 429 (MVCAM.A) | FITC | BD |
| Biotin | Streptavidin | PerCP-Cy5.5 | BD |
| Biotin | Streptavidin | APC | BD |
| Biotin | Streptavidin | eFluor®450 | eBioscience |

**Supplemental Figures**

**Figure Legends**

**Figure S1**: **Binding properties of POL5551 to CXCR4.** A0.01 cells overexpressing human CXCR4 were incubated with CXCL12, Plerixafor or POL5551 (1 µM for all) plus anti-CXCR4 antibody (Ab) clones 12G5 (extracellular loops) or 1D9 (N-terminus). CXCR4 Ab without agonist/antagonists (untreated) or isotypic control Ab (isotype) were used as positive and negative controls. Mean fluorescence intensity (arbitrary units) as percentage of the value from untreated cells is shown (mean±SEM, n=3).

**Figure S2: Kinetics of POL5551 mediated mobilization. A:** Assessment of POL5551 Pharmacokinetics. Plasma concentration of POL5551 following bolus injection in C57BL/6 mice. Blood was drawn at indicated time points post injection (i.p., 5 mg/kg) and analyzed for the presence of the compound (in grey, mean±SEM from 5 mice per time point). The CFU-C data (black curve) from Figure 2A are shown for comparison. **B**: Comparison of i.p. and i.v. administration route for POL5551. Male C57BL/6 mice received POL5551 (5 mg/kg) or NaCl (control) i.v. and blood was drawn at the indicated time points for CFU-C enumeration (mean±SEM, n=3 per group). CFU-C data from time-kinetics studies following i.p. injection of POL5551 in male C57BL/6 mice are shown for comparison (mean±SEM from 4-6 mice per time point for POL5551 and 3-9 mice per time point for control mice).

***p<0.001, **p<0.01, *p<0.05 compared to i.p route, ns, not significant

**Figure S3**: **Mobilization of mature cell subsets by POL5551**. **A**: Time-response of POL5551 mediated mobilization of WBCs. C57BL/6 mice received POL5551 (5 mg/kg) i.p. and blood was drawn at the indicated time points for blood count analysis (mean±SEM from 5 mice). **B**: Relative distribution of leukocytes in mobilized blood specimen. Blood was drawn before (baseline) and 4 hours after POL5551 injection i.p. (mean±SEM, n=5). Control mice received a standard regimen of G-CSF (standard regimen, mean±SEM, n=10) or a single injection of Plerixafor (5 mg/kg, i.p., blood sampling 1 hr post injection, mean±SEM, n=5). **p<0.01 compared to baseline, ns, not significant. **C**: Mobilization of T-, B-cells, monocytes and granulocytes. Blood composition was analyzed 4 hours after POL5551 injection (30 mg/kg, i.p. mean±SEM, n=6). Untreated (baseline, mean±SEM, n=6), G-CSF (standard regimen, mean±SEM, n=9) or Plerixafor (5 mg/kg, i.p., blood sampling 1 hr post injection, mean±SEM, n=6) treated mice served as controls. **D**: Assessment of mobilized T-cell subsets. Spleen cells from C57BL/6 mice mobilized with POL5551 (30 mg/kg, i.p., 4 hrs after injection, mean±SEM, n=7), G-CSF (standard regimen, mean±SEM, n=7), Plerixafor (5 mg/kg, i.p., 1 hr after injection, mean±SEM, n=6) or non-mobilized controls (baseline, mean±SEM, n=7) were analyzed with regard to the ratio of T-Helper cells (CD4+) to cytotoxic T-cells (CD8+) within the T-cell (CD3+) fraction.

**Figure S4: Dose-response data analysis.** Multiple regression analysis of the relationship between POL5551 dose (mg/kg) and the number of circulating CFU-C was performed. The best fit resulted from the MMF model as depicted in **A.** The corresponding curve is shown in **B.**

**Figure S5: CXCR4 surface expression on c-kit+ cells.** C57BL/6 mice received a single injection of POL5551 at the indicated dose or standard regimen of G-CSF. CXCR4 expression on mobilized c-kit+ was analyzed by flow cytometry in comparison to ssBM and ssPB ckit+. All specimens were evaluated relative to the samples stained with isotype control Ab. **A:** Percentage of CXCR4 positive cells. **B**: RMFI. (mean±SEM, n=5-10). ***p<0.001, **p<0.01

**Figure S6: RU Assay**

The frequency of repopulating units in POL5551 (30 mg/kg), Plerixafor (10 mg/kg), G-CSF (standard regimen), GCSF+POL5551 and G-CSF+Plerixafor mobilized blood was compared. Lethally irradiated recipients (n=4-9 per group) received transplants of 250,000 BM cells (CD45.2) together with a small volume of mobilized blood (CD45.1, n=2 donor mice per group) (6 µl for POL5551-, Plerixafor- or G-CSF-mobilized blood, 1.5 µl for blood mobilized with G-CSF+POL5551 or G-CSF+Plerixafor). Blood graft derived repopulating units were calculated for the 5 different sources according to B-cell and myeloid engraftment 12 weeks after transplantation (mean±SEM, n=3-9).

**Figure S7**: **Assessment of POL5551 in plasma and bone marrow**. Concentration of POL5551 in plasma and BM fluids following bolus injection in C57BL/6 mice. At indicated time points post injection (i.p. 5 mg/kg) plasma and marrow fluids were prepared and analyzed for the presence of the compound (in black and grey respectively, mean±SEM from 5 mice per time point).

**Suppl. Figure S1**

**Suppl. Figure S2**

**Suppl. Figure S3**

**Suppl. Figure S4**

**Suppl. Figure S5**

**Suppl. Figure S6**

**Suppl. Figure S7**

**Table S2: Immunophenotype of c-kit+ cells**

**C57BL/6 mice were mobilized with a bolus injection of POL5551 (5 or 30 mg/kg, i.p., n=5) or standard regimen of G-CSF (n=5). Saline treated animals (n=5) served as steady-state BM and PB donors. Blood and BM samples were collected (4 hrs after POL5551 injection, immediately after 9^th^ G-CSF dose or saline injection) and analyzed for surface expression of CD49d, CD49e, CD49f, CD29, CD106 and CD26 on c-kit+ cells. Percentage of positive cells was evaluated in comparison to isotype control. Mean fluorescence intensity was analyzed among c-kit+ cells.**

|  | **CD49d** | **CD49e** | **CD49f** | **CD29** | **CD26** | **VCAM1** |
| --- | --- | --- | --- | --- | --- | --- |
|  |  |  |  |  |  |  |
| **ssBM (% ckit +/- SEM)** | **98,2 +/-0,7** | **90,0 +/- 0,8** | **69,9 +/- 1,9** | **73,2 +/- 2,4** | **18,9 +/- 1,5** | **54,7 +/- 1,9** |
| **ssBM (RMFI +/- SEM)** | **4694 +/- 266** | **3022 +/- 80** | **1303 +/- 42,2** | **2119 +/- 53** | **570 +/- 26** | **1533 +/- 40** |
|  |  |  |  |  |  |  |
| **G-CSF (% ckit +/- SEM)** | **72,6 +/- 3,6** | **55,2 +/- 3,5** | **24,2 +/- 4,7** | **38,0 +/- 6,2** | **1,6 +/- 0,3** | **0,7 +/- 0,1** |
| **G-CSF (RMFI +/- SEM)** | **1179 +/- 58** | **772 +/- 39** | **642 +/- 104** | **1587 +/- 267** | **291 +/- 56** | **384 +/- 73** |
|  |  |  |  |  |  |  |
| **POL5551, 5 mg/kg (% ckit +/- SEM)** | **62,1 +/- 4,7** | **27,6 +/- 3,6** | **16,7 +/- 2,9** | **37,5 +/- 5,8** | **12,9 +/- 1,1** | **1,3 +/- 0,4** |
| **POL5551, 5 mg/kg (RMFI +/- SEM)** | **1909 +/- 192** | **912 +/- 98** | **487 +/- 50** | **1430 +/- 165** | **697 +/- 129** | **256 +/- 32** |
|  |  |  |  |  |  |  |
| **POL5551, 30 mg/kg (% ckit +/- SEM)** | **56,8 +/- 2,4** | **49,8 +/- 1,9** | **16,9 +/- 0,8** | **53,9 +/- 2,2** | **6,5 +/- 0,9** | **0,1 +/- 0,0** |
| **POL5551, 30 mg/kg (RMFI +/- SEM)** | **1602 +/- 114** | **2697 +/- 350** | **422 +/- 72** | **1692 +/- 208** | **534 +/- 145** | **110 +/- 5** |
|  |  |  |  |  |  |  |

Supplemental References

(1) Chang MK, Raggatt LJ, Alexander KA, Kuliwaba JS, Fazzalari NL, Schroder K, et al. Osteal tissue macrophages are intercalated throughout human and mouse bone lining tissues and regulate osteoblast function in vitro and in vivo. J Immunol 2008 Jul 15;181(2):1232-44.

(2) Wu B, Chien EY, Mol CD, Fenalti G, Liu W, Katritch V, et al. Structures of the CXCR4 chemokine GPCR with small-molecule and cyclic peptide antagonists. Science 2010 Nov 19;330(6007):1066-71.
